# Page kidney

**DOI:** 10.4103/0971-4065.59342

**Published:** 2009-10

**Authors:** A. Mathew, B. Brahmbhatt, R. Rajesh, G. Kurian, V. N. Unni

**Affiliations:** Department of Nephrology, Amrita Institute of Medical Sciences and Research Centre, Kochi, India

A 30-year-old male, a renal allograft recipient (donor was his sister), with baseline serum creatinine 1.2 mg%, was admitted 20 days after transplant surgery, with worsening of graft function (serum creatinine -1.8 mg%). Ultrasound revealed a normal graft with normal pelvicalyceal system. He underwent an ultrasound-guided graft kidney biopsy, using an automatic biopsy gun, under local anesthesia. Kidney biopsy showed features of Cyclosporin toxicity.

Following the procedure, he had macroscopic hematuria; Foley's catheter was inserted and urine gradually became clear. His graft function progressively worsened along with a drop in urine output and worsening of hypertension. On the third day following biopsy, the S. creatinine went up to 5.8 mg% and he required hemodialysis.

**Figure 1:**
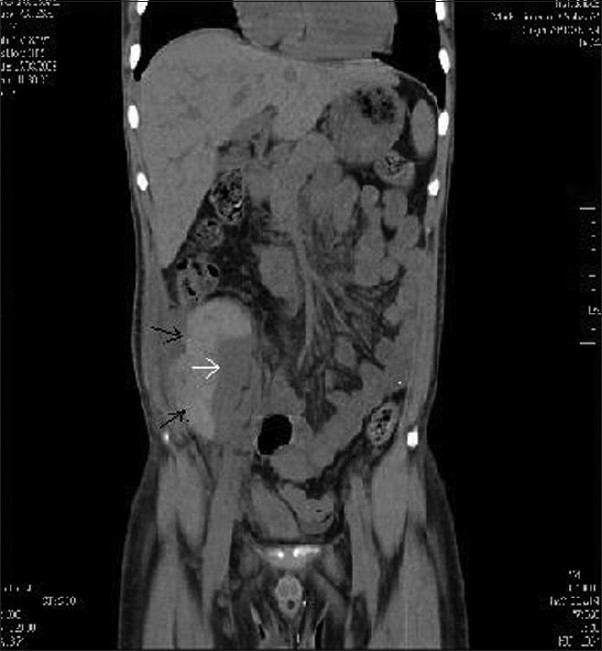
CT of the abdomen-(coronal section) showing sub capsular hematoma (black arrows) compressing the graft kidney (white arrow)

**Figure 2:**
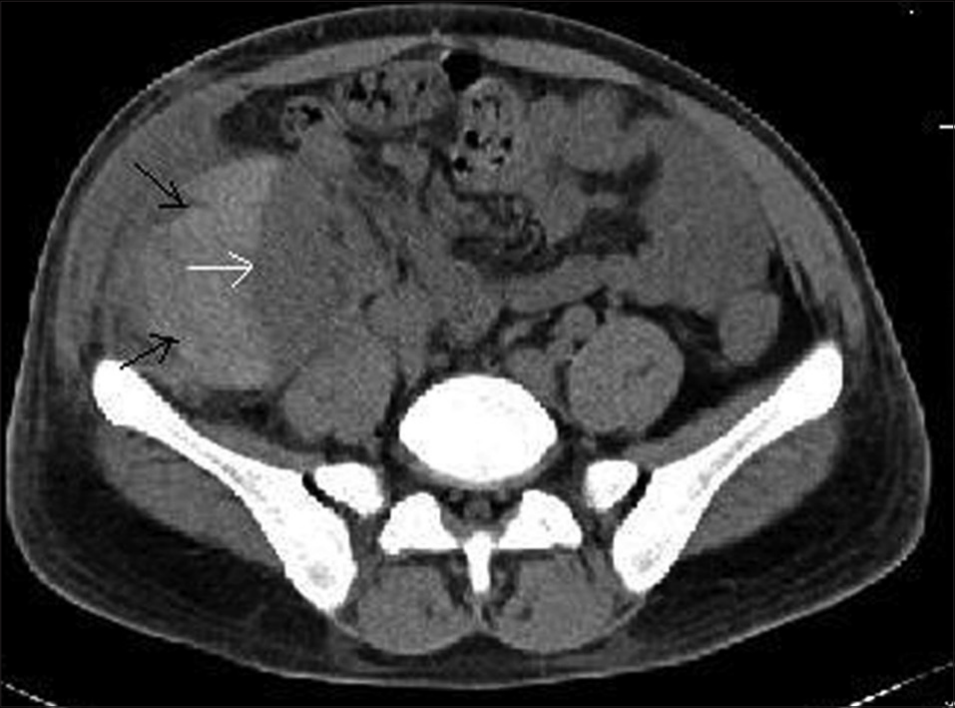
CT of the abdomen (transverse section) showing sub capsular hematoma (black arrows) compressing the graft kidney (white arrow)

Ultra sonogram showed a perigraft collection and a compressed pelvicaliceal system. A plain CT of abdomen showed a sub capsular hematoma (10 × 7 × 5 cm) compressing the graft kidney and its collecting system [Figures 1 and 2]. ‘Page kidney phenomenon’ was diagnosed and he underwent open drainage of hematoma under general anesthesia. His graft function and urine output improved. His blood pressures could be controlled with two antihypertensive medications. Repeat CT of abdomen showed normal graft with a small perigraft hematoma. On follow up, serum creatinine improved to 1.5 mg% by eighteenth day after drainage of the hematoma.

Renal allograft biopsy is an important investigative tool that provides information regarding the diagnosis, management and prognosis of graft dysfunction in the post transplant period. The procedure of graft biopsy is simple and safe in expert hands.[1] However, it does lead to complications in a small number of patients (5-8%). Perinephric hematomas account for 23-30% of these complications.[1] Oligoanuria following graft biopsy most commonly results from bleeding into the collecting system and formation of clots, which obstruct the ureter. Rarely, it can be due to a sub capsular hematoma compressing the renal parenchyma.

Page kidney is the external compression of a kidney usually caused by a sub capsular hematoma associated with high blood pressure and occasional renal failure.[2] It is named after Dr. Irvin Page (1901-1989) who first demonstrated in 1939 that wrapping cellophane tightly around animal kidneys could cause hypertension. In 1955, Page reported a case of a football player who suffered a blunt injury to the kidney producing a renal hematoma and renin-mediated hypertension. Thereafter, many cases of secondary hypertension associated with sub capsular or perinephric hematomas were reported. Although hypertension is the most common presenting disease, renal insufficiency can occur in the setting of a diseased contra lateral kidney, single functioning kidney or a renal allograft.

Anatomically, kidney is a poorly protected retroperitoneal organ that is surrounded by two envelopes. The first is the Gerota's fascia which is a large space and a large hematoma is needed in this space to compress the kidney. The second is the kidney capsule, which has a potential space that allows only small amount of blood to seep into it before compressing the kidneys and manifesting as hypertension or worsening of renal function. Sub capsular bleeding most commonly complicates renal biopsy and ESWL and usually does not cause hemodynamic instability.

The causes of Page kidney[2] may be divided into

- Bleeding
  - Due to trauma (American football and other contact sports, motor vehicle accidents)
  - Secondary to interventions (kidney biopsy, ESWL, post operative, sympathetic block)
  - Spontaneous bleeding (pancreatitis, Warfarin therapy, poly arteritis nodosa, tumors) and
- Nonbleeding causes
  - lymphoceles, large simple cysts, urinomas, retroperitoneal paraganglionomas

Various imaging modalities have been used to diagnose Page kidney. Ultrasound has the advantage of being cheap, easy to perform and noninvasive; but because it is highly operator dependent, it can miss small compressive sub capsular hematomas. Doppler evaluation also may give valuable clues toward the diagnosis of Page kidney. CT of the abdomen is the preferred modality as it is a noninvasive, readily accessible test, which can detect even very small hematomas.[3] MRI may be helpful in assessing the age of the hematomas and patency of renal blood vessels. Other investigations such as IVU, nuclear scan and renal arteriography are also helpful in evaluating a perirenal hematoma.

The aim of treatment of Page kidney is to spare the kidney and cure the hypertension. If hypertension is the major problem, without renal failure, potent antihypertensives including ACE-I may be sufficient and often nephrectomy can be avoided. If the hypertension persists, the hematoma is very large or if the renal function deteriorates, surgical evacuation of the hematoma is required. Percutaneous drainage is less invasive than open drainage but organized hematomas are non amenable to this mode of treatment. Success of laparoscopic evacuation may depend on the expertise of the surgeon.

Post biopsy anuria associated with a sub capsular hematoma in the renal allograft should raise a suspicion of Page kidney and an early recognition is warranted as immediate surgical decompression can salvage the allograft.[4]
